# Spirometry parameters used to define small airways obstruction in population-based studies: systematic review

**DOI:** 10.1186/s12931-022-01990-2

**Published:** 2022-03-21

**Authors:** Ben Knox-Brown, Octavia Mulhern, Johanna Feary, Andre F. S. Amaral

**Affiliations:** National Heart and Lung Institute, Imperial College London, 1B Manresa Road, London, SW3 6LR UK

**Keywords:** Respiratory epidemiology, Physiology, Spirometry, Small airways obstruction, Prevalence, Global

## Abstract

**Background:**

The assessment of small airways obstruction (SAO) using spirometry is practiced in population-based studies. However, it is not clear what are the most used parameters and cut-offs to define abnormal results.

**Methods:**

We searched three databases (Medline, Web of Science, Google Scholar) for population-based studies, published by 1 May 2021, that used spirometry parameters to identify SAO and/or provided criteria for defining SAO. We systematically reviewed these studies and summarised evidence to determine the most widely used spirometry parameter and criteria for defining SAO. In addition, we extracted prevalence estimates and identified associated risk factors. To estimate a pooled prevalence of SAO, we conducted a meta-analysis and explored heterogeneity across studies using meta regression.

**Results:**

Twenty-five studies used spirometry to identify SAO. The most widely utilised parameter (15 studies) was FEF_25–75_, either alone or in combination with other measurements. Ten studies provided criteria for the definition of SAO, of which percent predicted cut-offs were the most common (5 studies). However, there was no agreement on which cut-off value to use. Prevalence of SAO ranged from 7.5% to 45.9%. As a result of high heterogeneity across studies (I^2^ = 99.3%), explained by choice of spirometry parameter and WHO region, we do not present a pooled prevalence estimate.

**Conclusion:**

There is a lack of consensus regarding the best spirometry parameter or defining criteria for identification of SAO. The value of continuing to measure SAO using spirometry is unclear without further research using large longitudinal data.

*PROSPERO registration number* CRD42021250206

**Supplementary Information:**

The online version contains supplementary material available at 10.1186/s12931-022-01990-2.

## Introduction

Around 7% of the world population is estimated to be living with a chronic respiratory illness, with chronic obstructive pulmonary disease (COPD) and asthma being the most prevalent [1]. Since the late 1960s, the small airways of the lungs have been investigated as a site of interest in early obstructive lung disease [2]. The small airways are those with a diameter of less than 2 mm and have been described as a silent zone, where disease states can go unnoticed for many years [3]. Studies have shown that the small airways contribute significantly to airflow obstruction in both COPD [4] and asthma [5]. Characterised by inflammation, hypersecretion of mucus and airway remodelling [6], small airways obstruction (SAO) has been shown to precede both emphysematous changes and reduction in traditional spirometric parameters in COPD [7, 8].

In the absence of a non-invasive gold standard method to assess SAO, spirometry is the most widely used on account of its relatively easy performance and simple measurement devices [9]. In 1972, the mid-maximal expiratory flow rate (MMEF) was proposed as the best spirometric parameter to identify SAO [10]. MMEF, widely known as FEF_25-75_, corresponds to the mean forced expiratory flow between 25 and 75% of the forced vital capacity (FVC). Its use is based on the hypothesis that the mid-late portion of the FVC reflects the airflow through the small airways, which are prone to expiratory collapse due to their lack of cartilaginous support. In more recent years, other spirometry parameters have been used to identify SAO, including forced expiratory flow rates at 25%, 50% and 75% of the FVC (FEF_25_, FEF_50_, FEF_75_), and forced expiratory ratios such as the forced expiratory volume in three seconds (FEV_3_) as a ratio of the FVC (FEV_3_/FVC) [11]. The lack of consensus over which spirometry parameter is best to identify SAO is compounded by the wide range of definitions of an abnormal result, with lower limits of normal (LLN) [12], percent predicted [13] and other arbitrary cut-offs [14], being used to diagnose isolated SAO in the presence of otherwise normal spirometry.

Unsurprisingly, there is debate in the scientific community as to the clinical significance of isolated SAO [15, 16]. Despite this, population-based studies have attempted to provide estimates of prevalence and associated risk factors [13, 17], as well as demonstrate its usefulness as a prognostic marker for future development of chronic respiratory disease, such as COPD [18]. There is, therefore, an argument that identification of those with isolated SAO in the general population is important, and may highlight an unrecognised potentially symptomatic, clinical population who are at risk of further lung function decline, and in whom intervention may be warranted. We conducted a systematic review to evaluate the consensus in the literature regarding the spirometry parameters used to identify SAO and the cut-offs used to define abnormal results. In addition, we evaluated the prevalence estimates and risk factors for SAO identified in population-based studies.

## Methods

### Search strategy and selection criteria

We adhered to our published study protocol [19]. We included population-based studies that were cross-sectional, cohort or case-cohort in design. Selection was restricted to adult populations (≥ 18 years), where at least one spirometry parameter was used to define SAO. Effort was made to translate all articles that were not in English. Studies were excluded if they were not based in the general population (for example occupational or hospital based) or if longitudinal studies had less than 1-year follow-up. We searched Medline (PubMed) and Web of Science from database inception to 1 May 2021. We also searched for grey literature using Google Scholar and reviewed reference lists of included studies. Literature search strategies for Medline (PubMed) and Web of Science used medical subject headings (MeSH) and text words related to selected spirometry parameters and derivations of the phrase SAO. The search strategy is fully described in Additional file 1: Table S1. Publications returned by the search were imported into the Covidence web-based software (www.covidence.org), which automatically removed duplicates. When two studies reported on the same group of subjects, full texts were reviewed and the study with the most complete data relating to our study outcomes was included. For example, if one study measured SAO but did not report prevalence and one measured SAO and reported prevalence, then the latter study was given preference. We used a complete dual review approach in which title and abstract screening as well as full-text screening were independently done by two reviewers (BKB and OM). Disagreements were resolved after discussion with a third reviewer (AFSA). If abstracts contained insufficient information, they were included in the full text screening.

### Quality assessment

Study quality was evaluated independently by two reviewers (BKB and OM) using the Newcastle–Ottawa scale for observational studies [20]. Cross-sectional studies received a score from 0 to 8 and cohort studies from 0 to 9. They were rated on selection, comparability and outcome, and assigned a rating of good, fair or poor according to predefined quality criteria (Additional file 1: Tables S2, S5 and S6). Disagreements were resolved after discussion with author AFSA.

### Data extraction and analysis

We created and completed data extraction forms using the Covidence software. Data extraction was conducted by two reviewers (BKB and OM), and discrepancies were resolved after re-reviewing the full texts and discussion with a third reviewer (AFSA). Data extraction included study characteristics, primary outcomes (spirometry parameter used to measure SAO and definition of an abnormal result), secondary outcomes (prevalence estimates for SAO and odds ratios for risk factors), and for longitudinal studies, we extracted the number of years of follow-up and data on lung function decline.

### Pooled prevalence estimates for SAO

Where not explicitly stated, prevalence estimates for SAO were calculated from the number of cases and total size of the study population. When studies used multiple parameters or criterion to identify SAO, we used a hierarchy of evidence table to decide which parameters to include (Additional file 1: Table S3). To pool the prevalence estimates from several studies, we conducted a meta-analysis using Stata, version 17 (StataCorp LLC, TX, USA), and the *metaprop* command. Prior to meta-analysis, prevalence estimates were transformed using the Freeman-Tukey double arcsine method to account for overestimation of result weight, which could occur due to the presence of prevalence estimates at either extreme (0 or 1). To conduct the meta-analysis, we used a DerSimonian-Laird random effects model, which incorporates a measure of heterogeneity across studies. We tested for heterogeneity by using the I^2^ statistic, with a value > 75% indicating considerable heterogeneity. To explore potential sources of heterogeneity, we built a random effects meta regression model that included spirometry parameter, definition of an abnormal result, WHO region and gross national income (GNI). We used a backward elimination procedure and kept the variables that were significant in the final model. Due to significant heterogeneity across studies, pooled estimates were suppressed to avoid reporting inaccurate estimates of prevalence of SAO. Meta-analysis on risk factors and lung function decline was not possible due to insufficient data available for extraction.

## Results

**Fig. 1:**
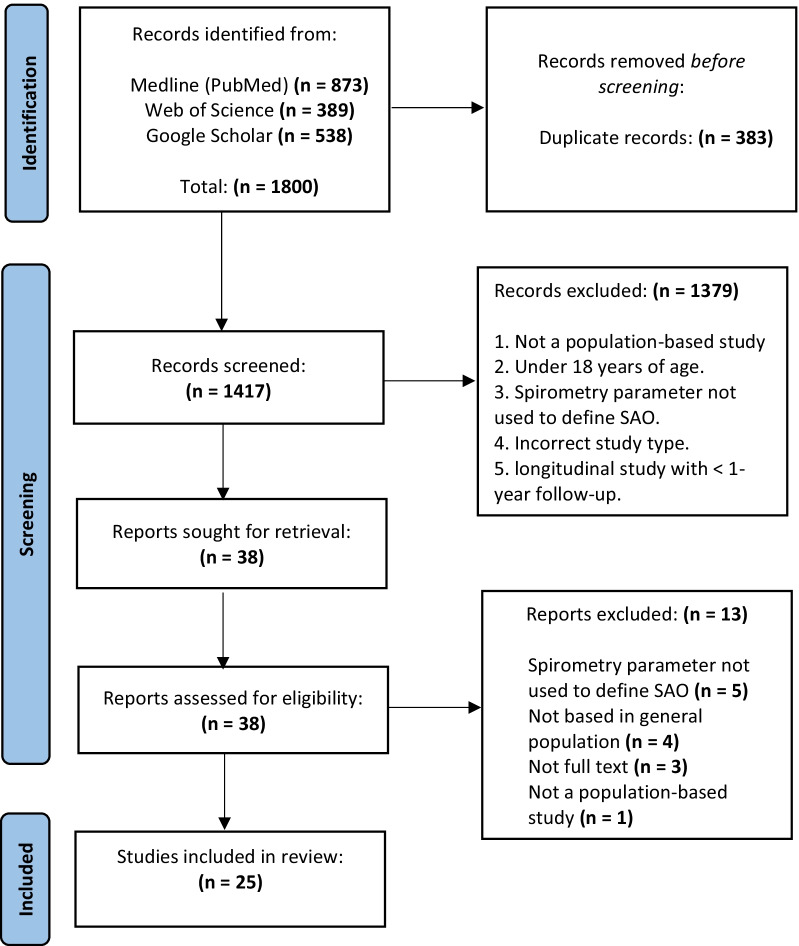
PRISMA flow diagram indicating study selection process

The search returned 1800 articles (full selection process is shown in Fig. 1) of which we identified 38 for full text review. One was in French and one was in Japanese, both of which were translated. We excluded 13 full texts, leaving 25 for inclusion. Publication dates ranged from 1979 to 2020. There were 18 cross-sectional, 6 cohort and 1 nested case–control study. All 25 studies used at least one parameter to identify SAO—these are described in full in Additional file 1: Table S4. Ten studies provided estimates for prevalence of SAO or contained data from which it could be calculated.

### Spirometry parameters used to measure SAO

**Fig. 2:**
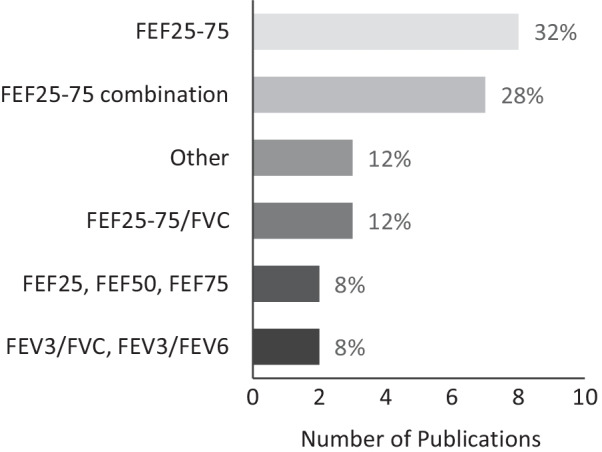
Spirometry parameters used to measure small airways obstruction in population-based studies. Other: Concavity index, FEF_50_/FEF_25_ and FET_25–75_

Across the 25 studies, 16 different spirometry parameters were used to identify SAO (Fig. 2). The majority (60%) used either FEF_25–75_ alone [21–28] or in combination with other parameters [13, 29–34]. Three studies used FEF_25–75_/FVC [35–37]. Two studies used FEF_25_, FEF_50_ or FEF_75_ either alone [38] or in combination [39]. One study used FEV_3_/FVC [40] and another used both FEV_3_/FVC and FEV_3_/FEV_6_. [12] Three studies used parameters that are not widely documented in the literature, forced expiratory time between 25 and 75% of the FVC (FET_25–75_) [41], concavity index [42] and forced expiratory flow at 50% of the FVC as a ratio of the forced expiratory flow with 25% of the FVC remaining (FEF_50_/FEF_25_) [14]. Twenty-one studies were of fair quality and 4 studies were of good quality (Additional file 1: Table S2).

### Definitions of an abnormal result

Ten studies presented criteria defining an abnormal result (Table 1), utilising eight separate methods. Three used percent predicted cut offs including: FEF_25–75_ < 75% [29], FEF_25–75_ < 67% [23], and FEF_50_ < 70% predicted [38]. Two used LLN for FEV_3_/FVC [12, 40]. One used concavity index above the upper limit of normal (ULN) [42], and two used other methods: FEF_50_/FEF_25_ > 4 [14]; and FEF_25–75_/VC < FEV_1_/VC [35]. A further two studies used a combination of criteria, one used 2 out of 3 of FEF_25–75_, FEF_50_ or FEF_75_ < 65% predicted [13], and one used one of FEF_25–75_ < 60% or FEF_25_ < 65% predicted.

**Table 1:** Summary of population-based studies estimating prevalence of small airways obstruction

Author, year	Study design	Population	Sample size	Age (years)	Sex	Spirometry Parameter(s) used to define SAO	Definition of abnormal result	Prevalence estimate(s) for SAO	Study quality (NOS)
Detels et al. 1979 [29]	Cross-sectional	Burbank and Lancaster, California, USA White (94%); black (1%); Spanish-surnamed (4%); other (1%)	N = 7974	18–65 +	F = 4126 (51.7%)	FEF_25–75_	< 75% predicted	> 18 years only Pre BD **21.4%**^**#**^	Fair
Wipf et al. 1982 [35]	Cohort Baseline and at 5 years follow-up	Geneva, Switzerland Current, former and never smokers	N = 272	18–50 +	F = 112, (41.2%)	FEF_25–75_/VC	FEF_25–75_/VC < FEV_1_/VC	Pre BD Baseline: **32.3%**	Fair
Marazzini et al. 1989 [32]	Cohort Baseline and 6 years	Italy, White-collar workers Smokers and non-smokers	N = 85	Mean (SD): 41.25 (7.4)	M = 85 (100%)	FEF_25-75_, FEF_25_ and CC	One of: FEF_25–75_ < 60%, FEF_25_ < 65% CC > 130% predicted FEV_1_ and VC > 80% predicted	Pre BD Baseline: **45.9%**^**#**^	Fair
Cullinan et al. 1997 [23]	Cross-sectional	Bhopal India Site of Union Carbide gas leak Never and ever smokers	Spirometry: N = 74 Total: N = 454	Mean (SD): 35.5 (3.2)	F = 44 (59.6%)	FEF_25-75_	< Lowest quartile (< 67% predicted)	Pre BD **25.7%**^**#**^	fair
Nemoto et al. 2011 [14]	Cross-sectional	Takahata Japan, participants of an annual health check Never and ever smokers	N = 2917	40–90	F = 1592 (54.6%)	FEF_50_/FEF_25_	> 4.0	Pre BD **36.5%**	Fair
Lam et al. 2012 [40]	Cross-sectional	Hong Kong Chinese Smokers only	N = 525	18–60 +	M = 525 (100%)	FEV_3_/FVC	< LLN	Pre BD **18.1%**	Fair
Chen et al. 2013 [38]	Nested case–control, with prevalence of SAO estimated from larger cohort	Moss Green, Huangqi Peninsula, Fujian, China Current former and never smokers	N = 2873 SAO: N = 216	Median (IQR): 50.5 (42–59) 50.5 (42–58)	F = 121 (56%) F = 240 (56%)	FEF_50_	< 70% predicted	Pre BD **7.5%**	Fair
Hansen et al. 2015 [12]	Cross-sectional	USA, NHANES-3 data Current smokers	N = 3508	20–79.9	F = 1571 (44.7%)	FEV_3_/FVC FEV_3_/FEV_6_	< LLN < LLN	Pre BD **16.3%** 16.6%	Fair
Johns et al. 2017 [42]	Cross-sectional Post BD only	BOLD study, Victoria and Tasmania, Australia Never and ever smokers	N = 890	Mean (SD): 58.7 (10.8)	F = 466 (52.4%)	FEF_25-75_ Central concavity Peripheral	FEV1/FVC > LLN < LLN > ULN > ULN	Post BD 5.4% 7.5% 9.8%	Fair
Xiao et al. 2020 [13]	Cross-sectional	CPH study, mainland China Covering all geographical regions Current, former and never smokers	N = 50,479	Mean (SD) 49.3 (13.8)	F = 29,213 (57.9%)	FEF_25–75_, FEF_50_ and FEF_75_ FEF_25–75_ FEF_50_ FEV_3_/FVC	2/3 < 65% predicted < LLN < 70% predicted < LLN With: FEV_1_ > 80% and FEV_1_/FVC > 0.7	Pre BD 28.5% Post BD 13.3% Pre BD: **26.9%** Pre BD: 36.5% Pre BD: 13.9%	Good

^#^Calculated from the information provided in a publication (cases/total population × 100). Prevalence estimates in **bold** indicate which estimates were used for meta-analysis. NOS: Newcastle–Ottawa scale, FEV_1_: forced expiratory volume in one second, FVC: forced vital capacity, FEF_25–75_: Mean expiratory flow rate between 25 and 75% of the FVC, FEF_25–75_/VC: Mean expiratory flow rate between 25 and 75% of the FVC as a ratio of the vital capacity. FEF_25_: Flow rate at 25% of FVC, CC: closing capacity, FEF_50_/FEF_25_: Forced expiratory flow at 50% of the FVC as a ratio or the forced expiratory flow with 25% of the FVC remaining, FEV_3_/FVC: forced expiratory volume in 3 s as a ratio of the FVC, FEV_3_/FEV_6_: forced expiratory volume in three seconds as a ratio of the forced expiratory volume in 6 s. FEF50: Forced expiratory flow at 50% of the FVC, SD: standard deviation, IQR: interquartile range. Pre BD: pre bronchodilator, Post BD: Post Bronchodilator (200 microg salbutamol), SAO: small airway obstruction, LLN: lower limit of normal, ULN: upper limit of normal

### Prevalence of small airways obstruction

Nine studies provided prevalence estimates for pre-bronchodilator SAO and are summarised in Table 1. Sex was split evenly across studies, except for two which recruited only males [32, 40]. In most studies, age ranged from 18 to 60 years, the smallest contained 74 participants [23] and the largest 50,479 participants [13]. Two studies were European [32, 35], 2 were from the Americas [12, 29], 4 from the Western Pacific [13, 14, 38, 40] and 1 from South-East Asia [23]. Five studies were from high-income countries [12, 14, 29, 32, 35] and 4 were from low- and middle-income countries (LMICs) [13, 23, 38, 40]. Estimates of SAO prevalence ranged from 7.5% [38] to 49.5% [32]. In studies that measured forced expiratory flow rates (FEF_25–75_, FEF_25_, FEF_50_ and FEF_75_), prevalence of SAO ranged from 7.5% [38] to 45.9% [32], and in studies that used forced expiratory ratios (FEF_25–75_/VC, FEF_50_/FEF_25_, FEV_3_/FVC and FEV_3_/FEV_6_) estimates ranged from 16.3% [12] to 36.5% [14]. When a percent predicted cut-off was used to define an abnormal result, prevalence estimates ranged from 7.5% [38] to 45.9% [32], and when LLN was used it ranged from 16.3% [12] to 26.9% [13]. In high-income countries, prevalence ranged from 16.3% [12] to 45.9% [32] and in LMICs, prevalence was between 7.5% [38] and 26.9% [13]. In males, prevalence ranged from 9.5% [38] to 45.9% [28] and in females from 7.5% [38] to 49.8% [35]. In those < 40 years old, prevalence of SAO ranged from 10.1% [12] to 45.9% [32], between 41 and 59 years old it was 15% [40] to 42.2% [35] and in those > 60 years old prevalence was between 25.3% [40] and 32.5% [29]. In never smokers, prevalence ranged from 19.8% [29] to 39.1% [32] and in ever smokers 24.3% [13] to 53.9% [32]. Two studies reported SAO prevalence post-bronchodilator, giving estimates of 5.4% [42] and 13.3% [13], respectively. Raw data for prevalence of SAO by subgroup can be found in Additional file 2.

**Fig. 3:**
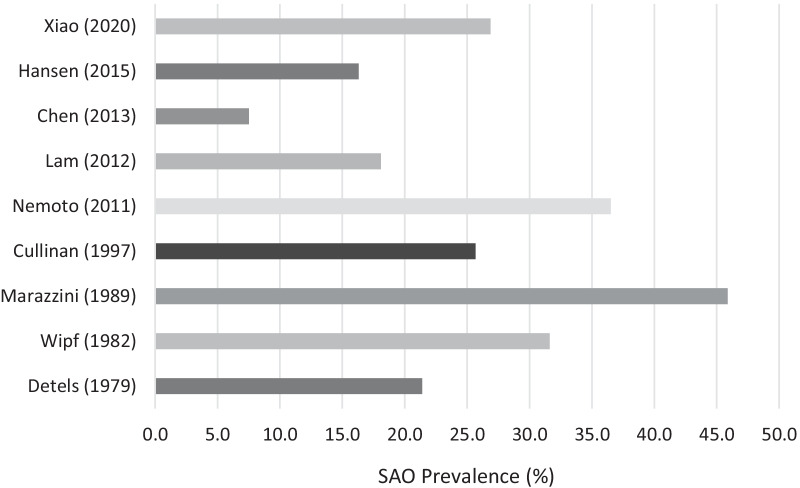
Prevalence of small airways obstruction, based on pre-bronchodilator spirometry, in nine studies

The prevalence of SAO across the nine studies with pre-bronchodilator measurements are displayed in Fig. 3. We did not report a pooled estimate due to marked heterogeneity across studies (I^2^ = 99.3%). The meta regression showed a significant effect of choice of spirometry parameter and WHO region on SAO prevalence. Results of the meta-regression are summarised in Table 2. These two variables accounted for 100% of the between-study variation. Using the Newcastle–Ottawa scale, 8 of the studies were scored as fair quality and one as good quality (Table 1).

**Table 2:** Meta-regression of covariates on prevalence of SAO

	Coefficient (% change in prevalence)	95% CI	*p* value
Spirometry parameter			
FEF_25–75_	(Ref)	**–**	**–**
FEF_25–75_/VC	− 14.3	− 38.6, 10.0	0.250
FEF_50_/FEF_25_	9.7	5.9, 13.4	< 0.001
FEV_3_/FVC	− 5.7	− 9.3, − 2.1	0.002
FEF_50_	− 19.3	− 23.1, − 15.6	< 0.001
WHO region			
Americas	(ref)	–	–
European	24.3	3.0, 45.6	0.025
Western Pacific	5.3	3.0, 7.5	< 0.001
South East Asia	4.1	− 18.7, 26.9	0.726

p < 0.05 = significant. FEF_25–75:_ Mean expiratory flow rate between 25 and 75% of the FVC, FEF_25–75_/VC: Mean expiratory flow rate between 25 and 75% of the FVC as a ratio of the vital capacity. FEF_50_: Forced expiratory flow rate at 50% of the FVC. FEF_50_/FEF_25_: Forced expiratory flow at 50% of the FVC as a ratio or the forced expiratory flow with 25% of the FVC remaining, FEV_3_/FVC: forced expiratory volume in 3 s as a ratio of the FVC

### Risk factors for small airways obstruction

Two Chinese studies reported adjusted odds ratios (OR) for the association of pre-bronchodilator SAO with risk factors (Table 3) [13, 38]. Both studies found that smoking was a significant risk factor for SAO, with Xiao et al. [13] showing that ever smokers were more likely to have SAO than never smokers (OR: 1.13, 95% CI 1.04–1.22), and Chen et al. [38] showing the same for heavy smokers (OR: 4.04, 95% CI 2.14–7.77). Chen et al. also found that exposure to second-hand smoke was associated with SAO (OR: 1.53, 95% CI 1.06–2.22) [38]. Only one of these studies found age and sex to be significant risk factors for SAO, with being > 70 years old associated with greater odds of having SAO compared to being 20–29 years old (OR: 2.41, 95% CI 2.13–2.72), and being female associated with increased odds of SAO (OR: 1.56, 95% CI 1.48–1.54) [13]. Both elevated body mass index (OR: 1.54, 95% CI 1.02–2.31) [38] and waist circumference (OR: 1.29, 95% CI 1.22–1.37) [13] were shown to be significant risk factors for SAO. Chen et al. [38] also reported a protective effect for exercising at least 30 min per day (OR: 0.31, 95% CI 0.20–0.48). Xiao et al. [13] found that biomass use (OR: 1.08, 95% CI 1.02–1.13) and high PM_2.5_ exposure (OR: 1.14, 95% CI 1.04–1.24) were associated with pre-bronchodilator SAO. Using the Newcastle–Ottawa scale, Chen et al. [38] was rated as fair, and Xiao et al. [13] rated as good quality (Additional file 1: Table S2).

**Table 3:** Risk factors for SAO

Study	Parameter used and definition of abnormal result	Prevalence of SAO	Covariates adjusted for	Risk factors for SAO (odds ratio and 95% confidence interval)
Chen et al. 2013 [38]	FEF_50_ < 70% predicted	7.5%	white cell count, diabetes, Total cholesterol, waist circumference, smoking index, second-hand smoke exposure, snoring, exercise	Diabetes: OR = 2.258 (1.042–4.890), p = 0.0039 High waist circumference: OR = 1.537 (1.023–2.310) p = 0.0039 Smoking Index > 600: OR = 4.044 (2.136–7.7656) p < 0.001 Second-hand smoke exposure: OR = 1.535 (1.060–2.224) p = 0.0023 Exercise for > 30 min per day: OR = 0.310 (0.200–0.482) p < 0.001
Xiao et al. 2020 [13]	2/3 of FEF_25–75_, FEF_50_ or FEF_75_ < 65% predicted With otherwise normal lung function: FEV_1_ > 80% and FEV_1_/FVC > 0.7	28.5%	male sex, age, rural residency, smoking exposure, smokers living in the home, biomass use, PM2·5 exposure, education level, history of tuberculosis, history of pneumonia or bronchitis during childhood, chronic cough during childhood, parental history of respiratory diseases, and BMI	Pre-BD Age: (reference 20–29): 30–39 OR 1.64 (1.48–1.82), 40–49 OR 2.22 (2.01–2.05), 50–59 OR 2.55 (2.31–2.81), 60–69 OR 2.54 (2.29–2.82), > 70 OR 2.41 (2.13–2.72), p < 0.0001 Post-BD: All associations with age remain significant (p < 0.0001) Pre-BD Gender: Female OR 1.56 (1.48–1.54) p < 0.0001 Post-BD Gender: Female OR 1.54 (1.43–1.64) p < 0.0001 Pre-BD Urban residence: Urban OR 1.07 (1.02–1.13) p = 0.0062 Post-BD Urban residence: Urban OR 1.13 (1.06–1.21) p = 0.0003 Pre-BD Education level: Primary school or less OR 1.11 (1.03–1.20) p = 0.0081 Post-BD Education level: Primary school or less OR 1.24 (1.11–1.28) p < 0.0001 Pre-BD Smoking: Ever OR 1.13 (1.04–1.22) p = 0.0040 Post-BD Smoking: Ever OR 1.18 (1.05–1.32) p = 0.0041 Pre-BD Biomass use: Yes OR 1.08 (1.02–1.03) p = 0.0060 Pre-BD: Annual mean PM2.5 exposure (per 25 mg/m^2^): 75 and above OR 1.14 (1.04–1.24) p = 0.0049. Post-BD: OR 1.27 (1.13–1.44) p = 0.0001 Pre-BD Chronic childhood cough (age < 14 years): Frequent OR 0.87 (0.77–1.0) p = 0.044 Pre-BD Parental history of respiratory diseases: Yes OR 1.08 (1.01–1.15) p = 0.024 Pre-BD BMI: Each 5 kg/m^2^ difference in BMI OR 1.29 (1.22–1.37) p < 0.0001 Post-BD BMI: Each 5 kg/m^2^ difference in BMI OR 1.37 (1.27–1.48) p < 0.0001

OR: odds ratio, FEF_50_ and FEF_75_: forced expiratory flow at 50% and 75% of the forced vital capacity (FVC), FEF_25–75_: Mean expiratory flow rate between 25 and 75% of the FVC, FEV_1_: forced expiratory volume in one second. BMI: body mass index. Statistically significant risk factors only extracted. Pre/post-BD: pre and post bronchodilator (200 mcg salbutamol)

Five studies assessed decline in FEF_25-75_ over time (Table 4). Two were from the UCLA cohort with follow-up after 5 years, [21, 30] two were from the SAPALDIA Study cohort with 11 years follow-up [25, 26], and one Italian study had 6 years of follow-up [32]. Taskin et al. [30] demonstrated that males who continued to smoke at follow-up had significantly greater decline in FEF_25–75_ (− 0.61 L/s) compared to those who quit prior to follow-up (− 0.39 L/s, p = 0.004). Three studies looked at the impact of pollution on FEF_25–75_ decline. Detels et al. [21] found that mean annual decline in FEF_25–75_ was significantly greater in the higher pollution Glendora region (− 93 ml/s) compared to the lower pollution Lancaster region of California (− 50 ml/s, p < 0.05). While the two studies from the SAPALIDA cohort showed that reduced exposure to PM10 was associated with a significant decrease (16%) in the annual rate of decline of FEF_25–75_, [26] and that certain gene polymorphisms attenuated annual rate of decline further [25]. Study quality was assessed as fair for two studies [30, 32], and good for three studies [21, 25, 26] (Additional file 1: Table S2).

**Table 4:** Decline in Spirometry parameters measuring SAO over time

Study	Parameter(s) used to measure SAO	Population	Years of follow-up	Outcomes
^#^Tashkin et al. 1984 [30]	FEF_25–75_	Lung function decline and smoking status. (UCLA study) N = 2400 Female: N = 1309, 54.5%. Mean age 45.6 years White non-Hispanic American	Lung function at baseline and after 5 years	5-year decline in FEF_25–75_ (L/s): Never smokers: Male = − 0.43 L/s Never smokers: Female = − 0.38 L/s Overall decline = − 0.41 L/s Former smokers at baseline: Male = − 0.36 L/s Former smokers at baseline: Female = − 0.33 L/s Overall decline = − 0.35 L/s Quitters at follow-up: Male = − 0.39 L/s Quitters at follow-up: Female = − 0.25 L/s Overall decline = − 0.32 L/s Continuing smokers: Male = − 0.61 L/s Continuing smokers: Female = − 0.46 L/s Overall decline = − 0.54 L Male smokers had significantly greater decline in FEF_25–75_ compared to quitters (p = 0.004); Females did not (p = 0.112) Total decline (all groups) = − 0.40 L/s
^#^Detels et al., 1987 [21]	FEF_25-75_	White, residents with non-Hispanic surnames in Glendora (high pollution) and Lancaster (Low pollution) who are never smokers. (UCLA study) Baseline: N = 1733, Female = 1102, 63.11% Retested: N = 932, Female = 611, 66% Age 19–59 years	Lung function at baseline and after 5.5 years 6 years in Lancaster and 5 years in Glendora	Mean (SE) annual decline in FEF_25–75_ in whole population = − 71.5 ml/s (12.25) Mean annual decline in FEF_25–75_ Lancaster = − 50 ml/s (8.5)—less polluted area Mean Annual decline in FEF_25–75_ Glendora = − 93 ml/s (8) p < 0.05 for comparison between populations Females: Lancaster = − 53 ml/s (7), Glendora = − 97 ml/s (6) Males Lancaster = − 47 ml/s (10), Glendora = − 89 ml/ (10)
Marazzini et al. 1989 [32]	FEF_25–75_, and FEF_25_	Male white-collar workers. Lung function decline and smoking status N = 85 Mean (SD) Age: 41.3 (7.4) years	Lung Function at baseline and after 6 years	Lung function decline over 6 years (L/s): Non-smokers with no SAO: FEF_25–75_ = − 0.22 L/s, p < 0.05 FEF_25_ = − 0.52 L/s, p < 0.05 Smokers with no SAO FEF_25–75_ = − 0.56 L/s, p < 0.05 FEF_25_ = − 0.54 L/s, p < 0.05 Non-smokers with SAO: FEF_25–75_ = − 0.34 L/s, p < 0.05 FEF_25_ = − 0.33 L/s, p < 0.05 Smokers with SAO: FEF_25_ = − 0.33 L/s, p < 0.05
^#^Downs et al. 2007 [26]	FEF_25–75_	Swiss Cohort Study on Air Pollution and Lung Diseases in Adults (SAPALDIA Study): PM10 exposure and lung function decline N = 4742 F = 2565, 54.1% Mean age (SD): 41.5 (11.3) years	Lung function performed at baseline and after 11 years	Decrease in annual rate of decline in FEF_25–75_ for 10 μg/m^3^ decrease in PM10 = 16% 11.3 ml/s (95%CI 4.3–18.2) p = 0.01 Decrease in annual rate of decline in FEF_25–75_ Interval Exposure of 109 μg/m^3^-year in PM10 = 14.0 ml/s (3.1–24.8) p = 0.01
^#^Curjuric et al. 2010 [25]	FEF_25–75_	SAPALDIA study: Gene polymorphisms and lung function decline due to PM10 reduction N = 4365 F = 2309, 52.9% Mean age (SD): 41.4 (11.3) years	Lung Function performed at baseline and after 11 years	Mean (SD) decline in FEF_25–75_ over 11 years = − 0.80 (0.7) L/s HMOX1 SNPs and related haplotypes attenuated the natural decline in FEF_25–75_ in the whole study sample by 5.9 mL·s^−1^ year^−1^ (95% CI − 0.5–12.3). p < 0.05. A 10 μg·m^−3^ reduction in PM10 significantly attenuated annual FEF_25–75_ decline by 15.3 mL·s^−1^ in the absence of HMOX1 haplotype ATC

^#^Studies based in same cohort of participants. FEF_25–75_: Mean expiratory flow rate between 25 and 75% of the FVC. FEF_25_: Forced expiratory flow rate at 25% of the FVC

## Discussion

We found 25 population-based studies that used spirometry parameters to assess outcomes relating to SAO. There was significant variation in diagnostic methods across studies, with 16 different spirometry parameters used. The most widely utilised parameter was FEF_25–75_ either alone, or in combination with other parameters. Only 10 studies (50%) gave diagnostic criteria for SAO, with 8 different methods being used. The most popular criterion used was a result being below a percent predicted cut-off. Arbitrary cut-offs between 60 and 75% predicted were selected without justification [32], or justified by referencing studies in clinical populations that themselves did not provide justification [13, 38]. Knudson and Lebowitz [43] showed in 1978 that the normal 95th percentile for FEF_25–75_ is actually closer to 56% predicted in those over 36 years old, and the benefits of using LLN as opposed to percent predicted cut-offs is well documented [44]. The methodological variation seen in this review is in agreement with a systematic review of 15 studies in adults with asthma, which reported 5 different spirometry parameters for diagnosing of SAO. [45].

We found 9 studies that provided pre-bronchodilator estimates for prevalence of SAO, ranging from 7.5% to 45.9%. Due to significant heterogeneity, we were unable to provide pooled estimates. From the meta-regression, we identified choice of spirometry parameter and WHO region as causes of this heterogeneity. The contribution of WHO region to heterogeneity across studies is not unexpected as the prevalence of obstructive lung disease varies across world regions [46]. We found that prevalence estimates where a percent predicted cut-off was used had a wider range than estimates where LLN was used. Similarly, FEV_3_/FVC was shown to have a narrower prevalence range than FEF_25–75_. In the study by Xiao et al. [13] the prevalence of SAO was 26.9% using FEF_25–75_ < LLN and 13.9% using FEV_3_/FVC < LLN, demonstrating the impact that choice of parameter can have on prevalence estimates. FEF_25–75_ has been shown to lack sensitivity, have large between subject variability in healthy populations, and potentially misestimate the prevalence of mild obstructive lung disease depending on the diagnostic criterion used [15, 47]. This may explain why some prevalence estimates are very high. In contrast, FEV_3_/FVC < LLN has been shown to be a sensitive measure of expiratory obstruction [47], associated with several indicators of mild lung injury [48]. Both methods rely on the accuracy of the measurement of the FVC, and provide overestimates of SAO when the FVC manoeuvre is terminated early. Even with these methodological limitations, spirometric measures of SAO have been shown to correlate well with markers of SAO taken from computed tomography (CT) scans in COPD [16]. However, spirometry has a significant advantage over other diagnostic methods such as thoracic CT scans and impulse oscillometry because devices are cheaper, more portable, and a wealth of potential data already exists in established population based studies.

Risk factors for SAO were found to be very similar to those for chronic airflow obstruction (CAO) [13, 38, 49], and included increasing age, previous smoking, passive smoke exposure, low education, and history of tuberculosis. Biomass fuel use for cooking and heating was found not to be a risk factor for post-bronchodilator SAO [13] in keeping with previous research on CAO [50], although available data are limited. However, in a single study, exposure to high annual levels of PM2.5 was associated with increased risk of post-bronchodilator SAO [13], contradicting previous research in populations with CAO [51]. In support of an association between SAO, smoking and PM2.5, the five included cohort studies reported a greater decline in FEF_25–75_ among current smokers, compared to never or former smokers [30], and a lesser decline in FEF_25–75_ in individuals exposed to lower levels of air particulates. A recent hospital-based study in adults, aged 40 years and above, has suggested that SAO might be an important predictor of COPD diagnosis several years later [18]. Therefore, the importance of isolated SAO at population level is its potential to be a modifiable precursor to future CAO.

The main limitation in the literature is related to the lack of agreement between studies as to which spirometry parameter or definition of an abnormal result to use to assess SAO. In addition, the observational design of included studies increases risk of bias. The overall quality of evidence included in this review was fair, this was largely due to selection bias caused by choice of study population [14, 23, 32, 40], and unsatisfactory response rate [12, 14, 23, 29, 38]. Additionally, in several studies assessment of SAO was not a primary outcome, meaning they were likely not sufficiently powered to draw conclusions. There was potential information bias in 6 studies [12, 14, 23, 29, 38, 40], which did not specify whether FEV_1_ and FEV_1_/FVC were normal in the populations diagnosed with SAO. In the three studies that did specify [13, 32, 35], arbitrary percent predicted cut-offs were used. This makes it hard to discern whether SAO was present due to existing obstructive lung disease or as its own clinical entity. There was also considerable variation in the spirometry reference equations used. Most studies used locally derived regression equations, while highly applicable to the population being studied, they are not transferable across regions. Additionally, only 50% of studies followed American Thoracic Society and European Respiratory Society (ATS/ERS) spirometry performance guidelines [52], meaning the quality of spirometry measurements in some studies could be questioned. Furthermore, most recent studies were based in the western pacific region, whereas studies providing prevalence estimates for SAO in Europe and the Americas were pre-2000 and likely not applicable to modern day. At review level, every effort was made to limit risk of bias. However, as the primary outcome of this study was which spirometry parameters are used to identify SAO, we may have missed publications which contained relevant data for prevalence estimates because they did not explicitly state that they were assessing SAO. In addition, as per the study protocol, we intended to assess certainty of evidence using the GRADE methodology for prevalence and risk factors for SAO [53]. However, as we could not present pooled estimates for either of these outcomes, and therefore draw firm conclusions, we could not comment on the certainty of evidence.

## Conclusions

This review highlights significant methodological inconsistencies in the measurement of SAO in population-based studies. Importantly, it highlights that researchers and clinicians should be cautious about continuing to use spirometry to identify SAO until a consensus is reached. Prevalence estimates derived from spirometry should not be used to inform policy while so many different diagnostic methods are being used. The significant association of SAO with risk factors such as smoking, air pollution, education level and age should not be ignored, but further examined in larger population-based studies. Future research should use LLN to estimate prevalence and risk factors for SAO with longitudinal follow-up, to determine whether those with SAO at baseline go on to develop obstructive lung conditions later in life.

## Supplementary Information

**Additional file 1. Table S1**. Search terms used within MEDLINE (PubMed) and Web of Science.** Table S2**. Quality assessment scores of selected studies.** Table S3**. Hierarchy of evidence ranking system.** Table S4**. Summary of different parameters used to assess small airways obstruction in population-based studies.** Table S5**. The modified Newcastle Ottawa scale for cross sectional studies.** Table S6**. The modified Newcastle-Ottawa scale for Cohort studies.

**Additional file 2.** Tables displaying raw data for prevalence of SAO by subgroup.

## Data Availability

All data generated or analysed during this study are included in this published article [and its additional information files].

## Abbreviations

COPD: Chronic obstructive pulmonary disease
CAO: Chronic airflow obstruction
SAO: Small airways obstruction
MMEF/FEF_25–75_: Mid-maximal expiratory flow rate
FEV_1_: Forced expiratory volume in 1 s
FVC: Forced vital capacity
FEV3: Forced expiratory volume in 3 s
FEV_1_/FVC: Forced expiratory volume in 1 s as a ratio of the forced vital capacity
FEV_3_/FVC: Forced expiratory volume in 3 s as a ratio of the forced vital capacity
FEV_3_/FEV_6_: Forced expiratory volume in 3 s as a ratio of the forced expiratory volume in 6 s
FEF_25, 50, & 75_: Forced expiratory flow rate at 25%, 50% and 75% of the forced vital capacity
LLN: Lower limit of normal
ULN: Upper limit of normal
MeSH: Medical subject heading
FET_25–75_: Forced expiratory time between 25 and 75% of the forced vital capacity
FEF_50_/FEF_75_: Forced expiratory flow rate at 50% of the forced vital capacity as a ratio of the forced expiratory flow rate at 75% of the forced vital capacity
IQR: Inter quartile range
CI: Confidence interval
SD: Standard deviation
NOS: Newcastle Ottawa scale
GRADE: Grading of recommendations, assessment, development and evaluation
WHO: World Health Organisation
CT: Computed tomography
Pre-BD: Pre bronchodilator
Post-BD: Post bronchodilator
BMI: Body mass index
ATS: American thoracic society
ERS: European respiratory society
PRISMA: Preferred Reporting Items for Systematic Reviews and Meta-Analyses
LMIC’s: Low/middle income countries
