# Supplementary material for: Spirometry parameters used to define small airways obstruction in population-based studies: systematic review
Source: Respir Res. 2022 Mar 21;23:67. doi: 10.1186/s12931-022-01990-2 (PMC8939095; doi:10.1186/s12931-022-01990-2)
Supplement: Supplementary file 1 — Additional file 1. Table S1. Search terms used within MEDLINE (PubMed) and Web of Science. Table S2. Quality assessment scores of selected studies. Table S3. Hierarchy of evidence ranking system. Table S4. Summary of different parameters used to assess small airways obstruction in population-based studies. Table S5. The modified Newcastle Ottawa scale for cross sectional studies. Table S6. The modified Newcastle-Ottawa scale for Cohort studies. [file 12931_2022_1990_MOESM1_ESM.pdf]

Appendix 1.

Table S1. Search terms used within MEDLINE (PubMed) and Web of Science.

Subject Heading	Search Terms Used
**Lung Function Parameters**	(Maximal midexpiratory flow rate OR FEF 25-75 Percent OR 25-75 Percent, FEF OR 25-75 Percent* FEF OR FEF 25 75 Percent OR FEF 25-75 Percent OR Percent, FEF 25-75 OR Percent*, FEF 25-75 OR MMFR OR Forced Expiratory Flow 025-075 Percent OR Forced Expiratory Flow 025 075 Percent) OR (forced expiratory flow OR mid-expiratory flow rate OR MMEF OR MMEF/FVC OR MEF50 OR MEF 50 OR MEF75 OR MEF 75 OR FEF25-75 OR FEF 50 OR FEF50 OR FEF 75 OR FEF75 OR FEV3/FVC OR FEV3 OR FEV3/FEV6).
	**AND**
**Small Airways Obstruction**	AND (Small airway* OR small airway* obstruction OR small airway* disease OR small airway* narrowing OR peripheral airway OR peripheral airway* disease OR peripheral airway* obstruction OR SAD OR small airway* dysfunction OR small airway* function OR small airway* limitation OR distal airway* OR distal airway* disease).

Grey literature strategy

After review of the literature, a Google Scholar search strategy was devised to identify grey literature. The strategy included the most frequently cited terms in relation to the spirometric assessment of small airways obstruction. These terms were adapted from the MEDLINE (PubMed) search strategy. The search was restricted to terms appearing in the title of the article and published between 1975-2021. These limits were introduced to restrict the number of results to only the most relevant publications [1].

“FEF25-75" OR "mid expiratory flow" OR MMEF OR MEF50 OR MEF75 OR FEF50 OR FEF75 OR FEV3/FVC OR FEV3/FEV6 AND "small airways obstruction" OR "small airways disease" OR "peripheral airways disease" OR "small airways dysfunction”

Table S2. Quality assessment scores of selected studies.

Study	Selection	Comparability	Outcome	Total	Rating
Detels et al, 1979 [2]	2	2	1	5	Fair
White et al, 1980 [3]	3	2	1	6	Fair
Tashkin et al, 1984 [4]	4	2	2	8	Fair
Marrero et al 1986 [5]	2	2	2	6	Fair
Detels et al, 1987 [6]	3	2	3	8	Good
Marazzini et al, 1989 [7]	2	2	2	6	Fair
Behera, 1997 [8]	3	2	1	6	Fair
Cullinan et al, 1997 [9]	2	2	2	6	Fair
Kiter et al, 2000 [10]	3	2	2	7	Fair
Matheson et al, 2006[11]	3	2	2	7	Fair
Downs et al, 2007 [12]	3	2	3	8	Good
Curjuric et al, 2010 [13]	3	2	3	8	Good
Chen et al, 2013 [14]	3	2	2	7	Fair
Abdel-Hamid, 2019 [15]	1	2	2	5	Fair
Al Khathlan et al, 2020 [16]	2	2	2	6	Fair
Havet et al, 2020 [17]	3	2	2	7	Fair
Xiao et al, 2020 [18]	4	2	2	8	Good
Wipf et al, 1982 [19]	3	1	2	6	Fair
Tager et al, 2005 [20]	2	2	2	6	Fair
Nemoto et al 2011 [21]	3	2	2	7	Fair
Lam et al, 2012 [22]	1	2	2	5	Fair
Hansen et al, 2015 [23]	2	2	2	6	Fair
Cox et al, 2020 [24]	3	2	2	7	Fair
Brown et al, 2005 [25]	2	2	2	6	Fair
Johns et al, 2017 [26]	2	2	2	6	Fair

Scores based on Newcastle-Ottawa Scale for cohort studies and adapted version for cross-sectional studies[27]. Scores out of a maximum of 8 for cross-sectional studies and 9 for cohort. Each item received a maximum of one star according scale shown in tables S5 and S6. Score quality rating was based on total number of stars received. Good quality: 2 in selection domain AND 3 in outcome domain. Fair quality: 1 star in selection domain OR 2 stars in outcome domain. Poor quality: If 0 stars in selection criteria OR 0-1 stars in outcome criteria.

Table S3. Hierarchy of evidence ranking system

Domain	Hierarchy of evidence
**Measurement of small airways obstruction using Spirometry**	FEF_{25-75} <LLN or FEV_3/FVC <LLN
	Other Spirometry parameter <LLN
	FEF_{25-75} < % predicted cut-off
	FEF_{50}, FEF_{25}, FEF_{75} <% Predicted
	Other

The rationale to place studies that used the LLN at the top is based on Miller et al. 2011, who showed that percent predicted cut-offs can misclassify the presence of airflow obstruction using FEV_1 and FEV_1/FVC.[28] FEF_{25}, FEF_{50} and FEF_{75} are at the bottom of the table as few studies have investigated these parameters. Table moves from highest to lowest strength of evidence. LLN: lower limit of normal, FEF_{25-75}: mean expiratory flow between 25% and 75% of the forced vital capacity (FVC), FEV_3/FVC: forced expiratory volume in three seconds as a ratio of the FVC, FEF_{25}, $_{50,}$ and $_{75}$: forced expiratory flow at 25%, 50% and 75% of the FVC.

Table S4. Summary of different parameters used to assess small airways obstruction in population-based studies.

Group	Author, year	Spirometry parameter(s) used to measure SAO	Primary study outcome
Forced expiratory flow rates	Detels et al, 1979 [2]	FEF_{25-75} and FEF_{50-75}	Lung function in high vs low pollution areas.
	White et al, 1980 [3]	FEF_{25-75} and FEF_{75-85}	Impact of passive smoking on lung function.
	Tashkin et al, 1984 [4]	FEF_{25-75}, FEF_{25}, FEF_{50} and FEF_{75}	Impact of smoking cessation on lung function.
	Marrero et al 1986 [5]	Vmax50% and Vmax25%	Discriminating power of different lung function measurements.
	Detels et al, 1987 [6]	FEF_{25-75}	Change in lung function over time.
	Marazzini et al, 1989 [7]	MMEF, Vmax25	Rate of lung function decline over time.
	Behera, 1997 [8]	FEF_{25-75}	Impact of common domestic fuels on lung function.
	Cullinan et al, 1997 [9]	FEF_{25-75}	Impact of Bhopal gas leak on the development airways disease
	Kiter et al, 2000 [10]	FEF_{25-75} FEF_{25}, FEF_{50}	Impact of water-pipe smoking on lung function
	Matheson et al, 2006[11]	FEF_{25-75}	Impact of gene polymorphisms on lung function.
	Downs et al, 2007 [12]	FEF_{25-75}	Impact of PM_{10} on age related decline in lung function.
	Curjuric et al, 2010 [13]	FEF_{25-75}	Impact of gene polymorphisms and PM_{10} on lung function.
	Chen et al, 2013 [14]	FEF_{50}	Prevalence and risk factors for SAO
	Abdel-Hamid, 2019 [15]	FEF_{25-75}	Impact of obesity of lung function.
	Al Khathlan et al, 2020 [16]	FEF_{25-75}	Impact of adiposity markers on lung function.
	Havet et al, 2020 [17]	FEF_{25-75} and FEF_{75}	Impact of outdoor air pollution on lung function
	Xiao et al, 2020 [18]	FEF_{25-75} FEF_{50}, FEF_{75}	Prevalence and risk factors for SAO
Forced expiratory ratios	Wipf et al, 1982 [19]	FEF_{25-75}/FVC	Change in lung function over time
	Tager et al, 2005 [20]	FEF_{25-75}/FVC	Impact of lifetime ozone exposure on lung function
	Nemoto et al 2011 [21]	FEF_{50}/FEF_{25}	Impact of smoking on SAO
	Lam et al, 2012 [22]	FEV_3/FVC	Detection of airflow obstruction
	Hansen et al, 2015 [23]	FEV_3/FVC, FEV_3/FEV_6	Define LLN for measures of SAO
	Cox et al, 2020 [24]	FEF_{25-75}/FVC, $FEF_{25,50,75}$/FVC,	Generation of reference equations.
Other	Brown et al, 2005 [25]	FET_{25-75}% (s)	Generation of reference equations
	Johns et al, 2017 [26]	Concavity index	Detection of SAO vs FEF_{25-75}

Abbreviations: FVC: forced vital capacity, FEF_{25-75}: mean expiratory flow between 25% and 75% of the FVC, FEF_{25-75}/VC: mean expiratory flow rate between 25% and 75% of the FVC as a ratio of the vital capacity. FEF_{50}/FEF_{25}: forced expiratory flow at 50% of the FVC as a ratio or the forced expiratory flow with 25% of the FVC remaining, FEV_3/FVC: forced expiratory volume in three seconds as a ratio of the FVC, FEV_3/FEV_6: forced expiratory volume in three seconds as a ratio of the forced expiratory volume in six seconds. $FEF_{25,\ 50,\ 75}$: forced expiratory flow at 25%, 50% and 75% of the FVC, FET_{25-75}%: forced expiratory time. SAO: small airway obstruction, LLN: lower limit if normal, PM_{10} particulate matter with diameter less than 10mm.

Table S5. The modified Newcastle Ottawa scale for cross sectional studies

**Selection**				**Comparability**	**Outcome**	
Representativeness of the sample	Sample size	Ascertainment of exposure	Non-respondents	Different outcome groups are comparable, based on the study design or analysis. Confounding factors are controlled.	Assessment of outcome	Was follow-up long enough for outcomes to occur
a) Truly representative of the average population in the community *<br><br>b) Somewhat representative of the average population in the community *<br><br>c) Selected group of users (e.g. smokers, non-smokers)<br><br>d) No description of the derivation of the cohort	a) Justified and satisfactory. *<br><br>b) Not justified.	a) Validated measurement tool. *<br><br>b) Non-validated measurement tool, but the tool is available or described. *<br><br>c) No description of the measurement tool.	a) Comparability between respondents and non-respondents characteristics is established, and the response rate is satisfactory. *<br><br>b) The response rate is unsatisfactory, or the comparability between respondents and non-respondents is unsatisfactory.<br><br>c) No description of the response rate or the characteristics of the responders and the non-responders.	The study controlled for the most important factor (smoking history). *<br><br>b) The study control for any additional factor (e.g. age, gender, ethnicity, education level, income).	a) Independent blind assessment *<br><br>b) Record linkage *<br><br>c) Self report<br><br>d) No description	a) The statistical test used to analyse the data is clearly described and appropriate, and the measurement of the association is presented, including confidence intervals and the probability level (p value). *<br><br>b) The statistical test is not appropriate, not described or incomplete

Table S6. The modified Newcastle-Ottawa scale for Cohort studies

Selection				Comparability	Outcome		
Representativeness of the exposed cohort	Selection of the non-exposed cohort	Ascertainment of exposure	Demonstration that outcome of interest was not present at start of study.	Comparability of cohorts on the basis of the design or analysis	Assessment of outcome	Was follow-up long enough for outcomes to occur	Adequacy of follow up of cohorts
a) Truly representative of the average population in the community studied *<br><br>b) Somewhat representative of the average population *<br><br>c) Selected group of users (e.g. smokers, non-smokers)<br><br>d) No description	a) Drawn from the same community as the exposed cohort *<br><br>b) Drawn from a different source<br><br>c) No description of the derivation of the non-exposed cohort	a) Secure record (e.g. clinical records) *<br><br>b) Structured interview *<br><br>c) Written self-report<br><br>d) No description	a) Yes *<br><br>b) No	a) The study controlled for the most important factor (smoking history). *<br><br>b) The study control for any additional factor (e.g. age, gender, ethnicity, education level, income).	a) Independent blind assessment *<br><br>b) Record linkage *<br><br>c) Self report<br><br>d) No description	a) Yes (>1 year) *<br><br>b) No	a) Complete follow up - all subjects accounted for *<br><br>b) Subjects lost to follow up unlikely to introduce bias (lost to follow up < 10 %)<br><br>c) Follow up rate < 10 % and no description of those lost<br><br>d) No statement

References

1. Haddaway NR, Collins AM, Coughlin D, et al. The Role of Google Scholar in Evidence Reviews and Its Applicability to Grey Literature Searching. *PLoS One* 2015;10(9):e0138237. doi: 10.1371/journal.pone.0138237 [published Online First: 2015/09/18]
2. Detels R, Rokaw SN, Coulson AH, et al. The UCLA population studies of chronic obstructive respiratory disease. I. Methodology and comparison of lung function in areas of high and low pollution. *Am J Epidemiol* 1979;109(1):33-58. doi: 10.1093/oxfordjournals.aje.a112658
3. White JR, Froeb HF. Small-airways dysfunction in nonsmokers chronically exposed to tobacco smoke. *N Engl J Med* 1980;302(13):720-3. doi: 10.1056/nejm198003273021304
4. Tashkin DP, Clark VA, Coulson AH, et al. The UCLA population studies of chronic obstructive respiratory disease. VIII. Effects of smoking cessation on lung function: a prospective study of a free-living population. *Am Rev Respir Dis* 1984;130(5):707-15. doi: 10.1164/arrd.1984.130.5.707
5. Marrero O, Beck GJ, Schachter EN. Discriminating power of measurements from maximum expiratory flow-volume curves. *Respiration* 1986;49(4):263-73. doi: 10.1159/000194889
6. Detels R, Tashkin DP, Sayre JW, et al. The UCLA population studies of chronic obstructive respiratory disease. 9. Lung function changes associated with chronic exposure to photochemical oxidants; a cohort study among never-smokers. *Chest* 1987;92(4):594-603. doi: 10.1378/chest.92.4.594
7. Marazzini L, Cavigioli G, Mastropasqua B, et al. FEV1 decline in asymptomatic young adults: relationships with some tests of small airways function. *Eur Respir J* 1989;2(9):817-21.
8. Behera D. An analysis of effect of common domestic fuels on respiratory function. *Indian J Chest Dis Allied Sci* 1997;39(4):235-43.
9. Cullinan P, Acquilla S, Dhara VR. Respiratory morbidity 10 years after the Union Carbide gas leak at Bhopal: A cross sectional survey. *Bmj-British Medical Journal* 1997;314(7077):338-42. doi: 10.1136/bmj.314.7077.338
10. Kiter G, Uçan ES, Ceylan E, et al. Water-pipe smoking and pulmonary functions. *Respir Med* 2000;94(9):891-4. doi: 10.1053/rmed.2000.0859
11. Matheson MC, Ellis JA, Raven J, et al. Association of ILS, CXCR2 and TNF-alpha polymorphisms and airway disease. *Journal of Human Genetics* 2006;51(3):196-203. doi: 10.1007/s10038-005-0344-7
12. Downs SH, Schindler C, Liu LJ, et al. Reduced exposure to PM10 and attenuated age-related decline in lung function. *N Engl J Med* 2007;357(23):2338-47. doi: 10.1056/NEJMoa073625
13. Curjuric I, Imboden M, Schindler C, et al. HMOX1 and GST variants modify attenuation of FEF25–75% decline due to PM10 reduction. *European Respiratory Journal* 2010;35(3):505-14.
14. Chen YS, Li XQ, Li HR, et al. Risk Factors for Small Airway Obstruction among Chinese Island Residents: A Case-Control Study. *PLoS One* 2013;8(7) doi: 10.1371/journal.pone.0068556
15. Abdel-Hamid HEM. Impulse oscillometry may be of value in detecting early effects of obesity on airway resistance. *Egyptian Journal of Chest Diseases and Tuberculosis* 2019;68(1):96-101. doi: 10.4103/ejcdt.ejcdt_106_18
16. Al Khathlan N, Salem AM. The Effect of Adiposity Markers on Fractional Exhaled Nitric Oxide (FeNO) and Pulmonary Function Measurements. *International Journal of General Medicine* 2020;13:955-62. doi: 10.2147/ijgm.S280395
17. Havet A, Hulo S, Cuny D, et al. Residential exposure to outdoor air pollution and adult lung function, with focus on small airway obstruction. *Environ Res* 2020;183:109161. doi: 10.1016/j.envres.2020.109161
18. Xiao D, Chen Z, Wu S, et al. Prevalence and risk factors of small airway dysfunction, and association with smoking, in China: findings from a national cross-sectional study. *Lancet Respir Med* 2020;8(11):1081-93. doi: 10.1016/s2213-2600(20)30155-7
19. Wipf R, Stinghe R, Perrin J. [First results of a longitudinal survey upon the small airways disease]. *Poumon Coeur* 1982;38(2):85-90.
20. Tager IB, Balmes J, Lurmann F, et al. Chronic exposure to ambient ozone and lung function in young adults. *Epidemiology* 2005;16(6):751-9. doi: 10.1097/01.ede.0000183166.68809.b0
21. Nemoto T, Shibata Y, Osaka D, et al. Impact of cigarette smoking on maximal expiratory flows in a general population: the Takahata study. *Intern Med* 2011;50(21):2547-55. doi: 10.2169/internalmedicine.50.5948
22. Lam DCL, Fong DYT, Yu WC, et al. FEV3, FEV6 and their derivatives for detecting airflow obstruction in adult Chinese. *The International journal of tuberculosis and lung disease* 2012;16(5):681-86.
23. Hansen JE, Porszasz J, Casaburi R, et al. Re-Defining Lower Limit of Normal for FEV1/FEV6, FEV1/FVC, FEV3/FEV6 and FEV3/FVC to Improve Detection of Airway Obstruction. *Chronic Obstructive Pulmonary Diseases-Journal of the Copd Foundation* 2015;2(2):94-102. doi: 10.15326/jcopdf.2.2.2014.0144

24. Cox CA, Vonk JM, Kerstjens HAM, et al. Predicted values for the forced expiratory flow adjusted for forced vital capacity, a descriptive study. *Erj Open Research* 2020;6(4) doi: 10.1183/23120541.00426-2020
25. Brown LK, Miller A, Pilipski M, et al. Forced midexpiratory time: reference values and the effect of cigarette smoking. *Lung* 1995;173(1):35-46. doi: 10.1007/bf00167599
26. Johns DP, Das A, Toelle BG, et al. Improved spirometric detection of small airway narrowing: concavity in the expiratory flow-volume curve in people aged over 40 years. *Int J Chron Obstruct Pulmon Dis* 2017;12:3567-77. doi: 10.2147/copd.S150280
27. Wells G SB, O'Connell D, Peterson J, Welch V, Losos M, Tugwell P. The Newcastle-Ottawa Scale (NOS) for assessing the quality of nonrandomised studies in meta-analyses http://www.ohri.ca/programs/clinical_epidemiology/oxford.asp2013 [
28. Miller MR, Quanjer PH, Swanney MP, et al. Interpreting lung function data using 80% predicted and fixed thresholds misclassifies more than 20% of patients. *Chest* 2011;139(1):52-9. doi: 10.1378/chest.10-0189 [published Online First: 2010/06/05]
