# Supplementary material for: Spirometry parameters used to define small airways obstruction in population-based studies: systematic review
Source: Respir Res. 2022 Mar 21;23:67. doi: 10.1186/s12931-022-01990-2 (PMC8939095; doi:10.1186/s12931-022-01990-2)
Supplement: Supplementary file 2 — Additional file 2. Tables displaying raw data for prevalence of SAO by subgroup. [file 12931_2022_1990_MOESM2_ESM.docx]

| **Author** | **Year** | **Cases** | **Population** | **Prevalence (%)** | **Spirometry Parameter Used** |
| --- | --- | --- | --- | --- | --- |
| Detels^1^ | 1979 | 1706 | 7974 | 21.4 | Mid-Expiratory Flow Rates |
| Marazzini^2^ | 1989 | 39 | 85 | 45.9 | Mid-Expiratory Flow Rates |
| Cullinan^3^ | 1997 | 19 | 74 | 25.7 | Mid-Expiratory Flow Rates |
| Chen^4^ | 2013 | 216 | 2873 | 7.5 | Mid-Expiratory Flow Rates |
| Xiao^5^ | 2020 | 14897 | 55420 | 26.9 | Mid-Expiratory Flow Rates |
| Wipf^6^ | 1982 | 86 | 272 | 31.6 | Mid-Expiratory Ratios |
| Nemoto^7^ | 2011 | 1065 | 2917 | 36.5 | Mid-Expiratory Ratios |
| Lam^8^ | 2012 | 95 | 525 | 18.1 | Mid-Expiratory Ratios |
| Hansen^9^ | 2015 | 572 | 3508 | 16.3 | Mid-Expiratory Ratios |

**Appendix 2.** Tables displaying Raw data for prevalence of SAO by subgroup

**Spirometry Parameter used to measure small airways obstruction (SAO)**

**Criterion used to define an abnormal result**

| **Author** | **Year** | **Cases** | **Population** | **Prevalence (%)** | **Definition of an abnormal result** |
| --- | --- | --- | --- | --- | --- |
| Detels^1^ | 1979 | 1706 | 7974 | 21.4 | < % predicted cut-off |
| Marazzini^2^ | 1989 | 39 | 85 | 45.9 | < % predicted cut-off |
| Cullinan^3^ | 1997 | 19 | 74 | 25.7 | < % predicted cut-off |
| Chen^4^ | 2013 | 216 | 2873 | 7.5 | < % predicted cut-off |
| Xiao^5^ | 2020 | 14897 | 55420 | 26.9 | <LLN |
| Lam^8^ | 2012 | 95 | 525 | 18.1 | <LLN |
| Hansen^9^ | 2015 | 572 | 3508 | 16.3 | <LLN |
| Wipf^6^ | 1982 | 86 | 272 | 31.6 | Other |
| Nemoto^7^ | 2011 | 1065 | 2917 | 36.5 | Other |

**WHO region**

| **Author** | **Year** | **Cases** | **Population** | **Prevalence (%)** | **WHO Region** |
| --- | --- | --- | --- | --- | --- |
| Detels^1^ | 1979 | 1706 | 7974 | 21.4 | Americas |
| Hansen^10^ | 2015 | 572 | 3508 | 16.3 | Americas |
| Marazzini^2^ | 1989 | 39 | 85 | 45.9 | European |
| Wipf^6^ | 1982 | 86 | 272 | 31.6 | European |
| Cullinan^3^ | 1997 | 19 | 74 | 25.7 | South East Asia |
| Chen^4^ | 2013 | 216 | 2873 | 7.5 | Western Pacific |
| Xiao^5^ | 2020 | 14897 | 55420 | 26.9 | Western Pacific |
| Lam^8^ | 2012 | 95 | 525 | 18.1 | Western Pacific |
| Nemoto^7^ | 2011 | 1065 | 2917 | 36.5 | Western Pacific |

| **Author** | **Year** | **Cases** | **Population** | **Prevalence (%)** | **World Bank Income Rating** |
| --- | --- | --- | --- | --- | --- |
| Detels^1^ | 1979 | 1706 | 7974 | 21.4 | High Income |
| Hansen^9^ | 2015 | 572 | 3508 | 16.3 | High Income |
| Marazzini^2^ | 1989 | 39 | 85 | 45.9 | High Income |
| Wipf^6^ | 1982 | 86 | 272 | 31.6 | High Income |
| Nemoto^7^ | 2011 | 1065 | 2917 | 36.5 | High Income |
| Cullinan^3^ | 1997 | 19 | 74 | 25.7 | Low-Middle Income |
| Chen^4^ | 2013 | 216 | 2873 | 7.5 | Low-Middle Income |
| Xiao^5^ | 2020 | 14897 | 55420 | 26.9 | Low-Middle Income |
| Lam^8^ | 2012 | 95 | 525 | 18.1 | Low-Middle Income |

**World Bank income rating**

| **Author** | **Year** | **Cases** | **Population** | **Prevalence (%)** | **Certainty of Evidence (GRADE)** |
| --- | --- | --- | --- | --- | --- |
| Detels^1^ | 1979 | 1706 | 7974 | 21.4 | Low Certainty |
| Hansen^9^ | 2015 | 572 | 3508 | 16.3 | Low Certainty |
| Cullinan^3^ | 1997 | 19 | 74 | 25.7 | Low Certainty |
| Wipf^6^ | 1982 | 86 | 272 | 31.6 | Moderate Certainty |
| Chen^4^ | 2013 | 216 | 2873 | 7.5 | Moderate Certainty |
| Xiao^5^ | 2020 | 14897 | 50479 | 29.5 | Moderate Certainty |
| Marazzini^2^ | 1989 | 39 | 85 | 45.9 | Very Low Certainty |
| Nemoto^7^ | 2011 | 1065 | 2917 | 36.5 | Very Low Certainty |
| Lam^8^ | 2012 | 95 | 525 | 18.1 | Very Low Certainty |

**Certainty of evidence using GRADE methodology**

**Publication year**

| **Author** | **Year** | **Cases** | **Population** | **Prevalence (%)** | **Year of Publication** |
| --- | --- | --- | --- | --- | --- |
| Detels^1^ | 1979 | 1706 | 7974 | 21.4 | 1975-2000 |
| Cullinan^3^ | 1997 | 19 | 74 | 25.7 | 1975-2000 |
| Wipf^6^ | 1982 | 86 | 272 | 31.6 | 1975-2000 |
| Marazzini^2^ | 1989 | 39 | 85 | 45.9 | 1975-2000 |
| Hansen^9^ | 2015 | 572 | 3508 | 16.3 | 2001-2015 |
| Chen^4^ | 2013 | 216 | 2873 | 7.5 | 2001-2015 |
| Nemoto^7^ | 2011 | 1065 | 2917 | 36.5 | 2001-2015 |
| Lam^8^ | 2012 | 95 | 525 | 18.1 | 2001-2015 |
| Xiao^5^ | 2020 | 14897 | 55420 | 26.9 | 2016- |

**Age group**

| **Author** | **Year** | **Cases** | **Population** | **Prevalence (%)** | **Age** |
| --- | --- | --- | --- | --- | --- |
| Wipf^6^ | 1982 | 50 | 183 | 27.3 | <40 Years |
| Marazzini^2^ | 1989 | 39 | 85 | 45.9 | <40 Years |
| Cullinan^3^ | 1997 | 19 | 74 | 25.7 | <40 Years |
| Lam^8^ | 2012 | 74 | 525 | 14.1 | <40 Years |
| Hansen^9^ | 2015 | 235 | 2322 | 10.1 | <40 Years |
| Xiao^5^ | 2020 | 2491 | 12818 | 19.4 | <40 Years |
| Wipf^6^ | 1982 | 38 | 90 | 42.2 | 41-60 Years |
| Lam^8^ | 2012 | 79 | 525 | 15.0 | 41-60 Years |
| Hansen^9^ | 2015 | 338 | 1186 | 28.5 | 41-60 Years |
| Xiao^5^ | 2020 | 7876 | 25036 | 31.5 | 41-60 Years |
| Detels^1^ | 1979 | 421 | 1293 | 32.6 | >60 Years |
| Lam^8^ | 2012 | 40 | 158 | 25.3 | >60 Years |
| Xiao^5^ | 2020 | 4034 | 12625 | 32.0 | >60 Years |

**Smoking status**

| **Author** | **Year** | **Cases** | **Population** | **Prevalence (%)** | **Smoking Status** |
| --- | --- | --- | --- | --- | --- |
| Detels^1^ | 1979 | 1044 | 5265 | 19.8 | Never Smoker |
| Wipf^6^ | 1982 | 18 | 51 | 35.3 | Never Smoker |
| Marazzini^2^ | 1989 | 18 | 46 | 39.1 | Never Smoker |
| Xiao^5^ | 2020 | 10896 | 36048 | 30.2 | Never Smoker |
| Detels^1^ | 1979 | 1515 | 5265 | 28.8 | Ever Smoker |
| Wipf^6^ | 1982 | 43 | 109 | 39.4 | Ever Smoker |
| Marazzini^2^ | 1989 | 21 | 39 | 53.8 | Ever Smoker |
| Xiao^5^ | 2020 | 3505 | 14431 | 24.3 | Ever Smoker |

| **Author** | **Year** | **Cases** | **Population** | **Prevalence (%)** | **Sex** |
| --- | --- | --- | --- | --- | --- |
| Wipf^6^ | 1982 | 333 | 1325 | 25.1 | Male |
| Marazzini^2^ | 1989 | 39 | 85 | 45.9 | Male |
| Lam^8^ | 2012 | 80 | 440 | 18.2 | Male |
| Chen^4^ | 2013 | 95 | 1001 | 9.5 | Male |
| Hansen^10^ | 2015 | 319 | 1937 | 16.5 | Male |
| Xiao^5^ | 2020 | 4855 | 21266 | 22.8 | Male |
| Wipf^6^ | 1982 | 793 | 1592 | 49.8 | Female |
| Chen^4^ | 2013 | 121 | 1606 | 7.5 | Female |
| Hansen^9^ | 2015 | 254 | 1571 | 16.2 | Female |
| Xiao^5^ | 2020 | 9546 | 29213 | 32.7 | Female |

**Sex**

**References**

1. Detels R, Rokaw SN, Coulson AH, et al. The UCLA population studies of chronic obstructive respiratory disease. I. Methodology and comparison of lung function in areas of high and low pollution. *Am J Epidemiol* 1979;109(1):33-58. doi: 10.1093/oxfordjournals.aje.a112658

2. Marazzini L, Cavigioli G, Mastropasqua B, et al. FEV1 decline in asymptomatic young adults: relationships with some tests of small airways function. *Eur Respir J* 1989;2(9):817-21.

3. Cullinan P, Acquilla S, Dhara VR. Respiratory morbidity 10 years after the Union Carbide gas leak at Bhopal: A cross sectional survey. *Bmj-British Medical Journal* 1997;314(7077):338-42. doi: 10.1136/bmj.314.7077.338

4. Chen YS, Li XQ, Li HR, et al. Risk Factors for Small Airway Obstruction among Chinese Island Residents: A Case-Control Study. *PLoS One* 2013;8(7) doi: 10.1371/journal.pone.0068556

5. Xiao D, Chen Z, Wu S, et al. Prevalence and risk factors of small airway dysfunction, and association with smoking, in China: findings from a national cross-sectional study. *Lancet Respir Med* 2020;8(11):1081-93. doi: 10.1016/s2213-2600(20)30155-7 [published Online First: 2020/07/01]

6. Wipf R, Stinghe R, Perrin J. [First results of a longitudinal survey upon the small airways disease]. *Poumon Coeur* 1982;38(2):85-90.

7. Nemoto T, Shibata Y, Osaka D, et al. Impact of cigarette smoking on maximal expiratory flows in a general population: the Takahata study. *Intern Med* 2011;50(21):2547-55. doi: 10.2169/internalmedicine.50.5948

8. Lam DCL, Fong DYT, Yu WC, et al. FEV3, FEV6 and their derivatives for detecting airflow obstruction in adult Chinese. *The International journal of tuberculosis and lung disease* 2012;16(5):681-86.

9. Hansen JE, Porszasz J, Casaburi R, et al. Re-Defining Lower Limit of Normal for FEV1/FEV6, FEV1/FVC, FEV3/FEV6 and FEV3/FVC to Improve Detection of Airway Obstruction. *Chronic Obstructive Pulmonary Diseases-Journal of the Copd Foundation* 2015;2(2):94-102. doi: 10.15326/jcopdf.2.2.2014.0144

10. Hansen JE, Sun XG, Wasserman K. Discriminating measures and normal values for expiratory obstruction. *Chest* 2006;129(2):369-77. doi: 10.1378/chest.129.2.369 [published Online First: 2006/02/16]
